# Evaluation of molecular assays to detect *Leishmania donovani* in *Phlebotomus argentipes* fed on post-kala-azar dermal leishmaniasis patients

**DOI:** 10.1186/s13071-021-04961-6

**Published:** 2021-09-09

**Authors:** Md Anik Ashfaq Khan, Khaledul Faisal, Rajashree Chowdhury, Rupen Nath, Prakash Ghosh, Debashis Ghosh, Faria Hossain, Ahmed Abd El Wahed, Dinesh Mondal

**Affiliations:** 1 Institute of Animal Hygiene and Veterinary Public Health, University of Leipzig, An den Tierkliniken 43, 04103 Leipzig, Germany; 2 Nutrition and Clinical Services Division, International Centre for Diarrheal Disease Research Bangladesh, 1212 Dhaka, Bangladesh; 3 Laboratory Sciences and Services Division, International Centre for Diarrheal Disease Research Bangladesh, 1212 Dhaka, Bangladesh

**Keywords:** Post-kala-azar dermal leishmaniasis, Sand fly, Molecular assay, MinION sequencing, Leishmaniasis transmission

## Abstract

**Background:**

Post-kala-azar dermal leishmaniasis (PKDL) caused by *Leishmania donovani* (LD) is a skin disorder that often appears after treatment of visceral leishmaniasis (VL) patients. PKDL patients are potential reservoirs of LD parasites, which can initiate a new epidemic of anthroponotic VL. Therefore, host infectiousness to its sand fly vector is a critical factor for transmission, and its accurate estimation can facilitate control strategies. At present, conventional microscopy serves as the reference method to detect parasites in its vector. However, low sensitivity of microscopy can be a limiting factor.

**Methods:**

In this study, real-time quantitative PCR (LD-qPCR) and recombinase polymerase amplification (LD-RPA) assays were evaluated against microscopy for the detection of LD DNA extracted from live sand flies five days after controlled feeding on PKDL cases.

**Results:**

The sensitivity of LD-qPCR and LD-RPA assays were found to be 96.43 and 100%, respectively, against microscopy for the selected fed sand flies (*n* = 28), and an absolute specificity of both molecular tools for apparently unfed sand flies (*n* = 30). While the proportion of infectious cases among 47 PKDL patients was estimated as 46.81% as defined by microscopic detection of LD in at least one fed sand fly per case, LD-RPA assay evaluation of only the microscopy negative sand flies fed to those 47 PKDL cases estimated an even greater proportion of infectious cases (51.06%). In overall estimation of the infectious cases in retrospective manner, discordance in positivity rate was observed (*p* < 0.05) between LD-RPA (59.57%) assay and microscopy (46.81%), while LD-RPA had slightly better positivity rate than LD-qPCR (55.32%) as well.

**Conclusions:**

Considering the sensitivity, cost, detection time, and field applicability, RPA assay can be considered as a promising single molecular detection tool for investigations pertaining to LD infections in sand flies and/or host infectiousness in PKDL, while it can also be useful in confirmation of microscopy negative sand fly samples.

**Graphical abstract:**

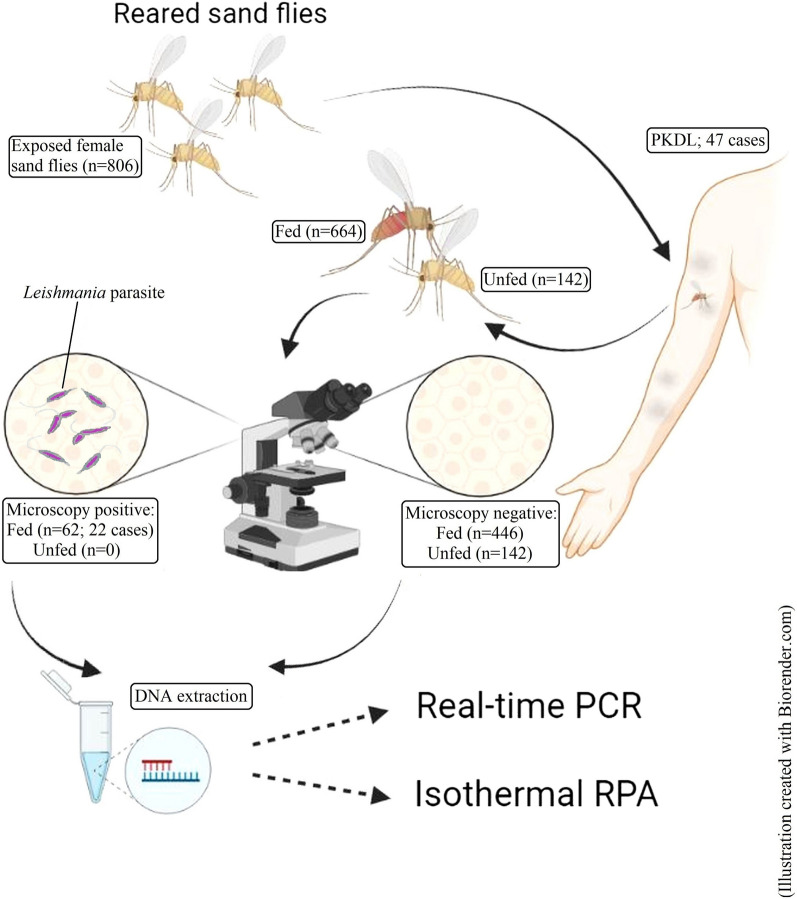

## Background

Post-kala-azar dermal leishmaniasis (PKDL) is an atypical dermatosis caused by *Leishmania donovani* (LD) infection, and develops as a sequel of leishmaniasis mostly after successful treatment of visceral leishmaniasis (VL). In Asia, development of PKDL has been reported to occur in a significant percentage (between 5–49%) of patients depending on the follow-up period (6 months to 5 years) after VL treatment [1, 2]. On the other hand, the rate of PKDL development is considered higher for the East African endemic foci; in Sudan, several studies suggest development of PKDL in about 50–60% of VL cases [3, 4]. PKDL patients are prone to parasite uptake if bitten by its sand fly vector, because typical PKDL manifestation is in the form of painless macular or papulo-nodular lesions, or a mix of both, that harbour parasites. This may play a major role in the transmission cycle of leishmaniasis [5]. In fact, it is thought to play a role in the recurrence of VL/kala-azar outbreaks in the endemic areas of South-East Asian countries, which is why the control of PKDL is among the priority objectives of the regional initiative for elimination of VL, known as the Kala-azar Elimination Programme (KAEP) [6]. In order to design an effective strategy to control PKDL mediated transmission, it is important to perform studies to elucidate the contribution of PKDL patients to sand fly infection with LD. The only accepted proof of host infectiousness so far is xenodiagnosis (XD), which entails demonstration of infection in laboratory-reared sand flies after feeding on a putative reservoir host [7]. The advantage of XD lies in its capability of utilizing the insect vector as biological culture medium to amplify an inoculum of living parasites even with very low parasite load, thus constituting irrefutable evidence of viable and transmissible LD infection. This information can be especially important to evaluate host and/or vector infectiousness status in epidemiological surveillance and to assess the effect of chemotherapeutics on the reservoir potential and their efficacy in treated cases of PKDL patients without invasive procedures to establish whether the infection has healed or not [8].

Since 1928, several studies from the Indian subcontinent, including two recent studies in Bangladesh, have assessed host infectivity by feeding uninfected sand flies on PKDL patients and subsequent measurement of infection rates [5, 9–11]. The current gold standard for parasite detection in sand flies is microscopic observation, which demonstrates viable and stable transfer of parasites. However, along with the chance of misinterpreting lower parasite load, microscopy is labour-intensive and requires expertise during eco-epidemiological surveys, which usually involve screening of a large number of vectors, and/or in XD studies conducted on a large number of cases and with large number of vector population. As a consequence, microscopic results may inappropriately estimate the infection rate as well as percentage of infectious population. In an alternative approach, we have shown in our previous XD study with quantitative polymerase chain reaction (qPCR) of skin punch biopsy that parasite load is associated with infectiousness of PKDL patients to sand fly and can be a promising surrogate or complementary marker of onward transmission potential [10]. Results in that study also suggest that LD-qPCR coincides considerably with microscopy for detection of LD parasites in sand flies. To improve the limited sensitivity of the direct observation, especially with low parasite numbers, PCR techniques have been increasingly applied for estimation of parasite infection rates in vectors and mammalian hosts [10, 12–15]. Moreover, molecular tools can also be very useful for the XD of *Leishmania* species whose natural vector(s) is unknown or not colonized, as parasites must be detected in the blood meal before defecation by the vector, which might be difficult using microscopy [16]. However, PCR/qPCR has limited applicability in remote field settings, where laboratory-bred or environmentally captured sand flies could be in regular need of monitoring for host infectiousness status or natural infection rate, respectively. In recent times, on the other hand, isothermal amplification-based assays have been attracting tremendous interest in diagnostic research for their field feasible nature along with high sensitivity and specificity [17]. For the detection of clinically important *Leishmania* species, several assays have been developed to date by using the isothermal platforms of recombinase polymerase amplification (RPA), loop-mediated isothermal amplification (LAMP), and nucleic acid sequence-based assay (NASBA) [18–20]. LAMP assay was found to be robust and sensitive in the detection of *Leishmania* species in sand fly specimens as well [21, 22]. Nevertheless, RPA offers several advantages over LAMP in terms of faster time to results, simpler primer design, longer target sequence, more tolerance to inhibitors, and dispensability of heating source [23, 24]. For the diagnosis of VL and PKDL in clinical samples, we previously developed an LD-RPA assay which showed absolute agreement with LD-qPCR results. Furthermore, to facilitate the use of the assay in field settings, two mobile suitcase laboratories incorporating nucleic acid extraction and detection systems were developed [19]. In the present study, we evaluated the performance of LD-qPCR and LD-RPA assays against microscopy for detection of LD in DNA samples extracted from sand flies that were fed on PKDL patients. Furthermore, we explored parasite load in fed sand fly samples that were negative in microscopy, and compared the overall positivity rates for infectiousness.

## Methods

Archived sand fly DNA samples as well as sand fly specimens from our previous study (Ethical Review Committee approved protocol no. PR-14010, International Centre for Diarrhoeal Disease Research, Bangladesh) were retrieved. In accordance with that study, the total number of PKDL cases with correspondence to which sand fly samples were obtained for this study was 47 [10]. Host positivity for infectiousness by microscopy was defined as the detection of LD promastigotes in at least 1 fly within the pool of flies that were fed on an individual host.

### Sample source and processing

The archived samples were generated from direct XD experiments [10], which were conducted as described previously [9]. Briefly, the participant placed exposed lesion site into a cage for 15 min; the cage contained 20 to 25 five-day-old female *Phlebotomus argentipes* and 5–10 male flies. Unfed flies were separated from fed flies with an aspirator; flies were kept for 5 days at 27 ºC, 85–95% humidity, and fed on a 30% sucrose diet. Flies still living 5 days after the XD feeding procedures were anesthetized with carbon dioxide/chloroform, placed in a drop of sterile phosphate-buffered saline (PBS) on a microscope slide, and decapitated with needles. The midgut was drawn out and placed in another PBS drop and examined under optical microscope at 40× to detect mobile promastigotes. Positive slides were Giemsa stained in a 1:9 PBS solution for confirmation of parasites and stored at room temperature. A total of 22 microscopy positive (Mic+) individual sand fly samples which represent all microscopy positive infectious PKDL cases were taken randomly from the positive sand fly pools of each case. Six more samples were then randomly taken from rest of the positive sand flies. Thirty microscopy negative (Mic−) and apparently unfed sand fly samples following XD experiments were included in the assessment of specificity of molecular tools. All of the selected sand fly specimens held on slides were subjected to DNA extraction separately (as discussed below). On the other hand, Mic− and apparently fed sand fly specimens were pooled together case-wise for all the PKDL cases (*n* = 47) and stored in 90% ethanol solution until DNA extraction. All samples were handled under sterile conditions to avoid cross-contamination.

### DNA extraction

DNA was extracted from stored Giemsa smeared slide specimens by using Qiagen kits. The process of DNA extraction from Giemsa stained sand fly tissue smear was optimized from a previously published protocol for blood smear content [25]. Briefly, 180 µl buffer ATL (Qiagen, Germantown, MD, USA) and 20 µl proteinase K (20 mg/ml; Qiagen) were combined in a 1.5 ml microtube for each midgut tissue smear. Using a pipette, 100 µl of the buffer ATL/proteinase K lysis mix was dispersed onto a slide for 10 s, and then a sterile sharp blade was used to scrap the slide for dissociation of visible smear residues. The same pipette tip was used to bring the liquid from blade and slide together and then transfer it back into the 1.5 ml microtube with the remaining ATL buffer/proteinase K mix. DNA from pooled sand fly samples were extracted as described [10] and the archived DNA samples were retrieved for this study. The extraction procedure involved centrifugation and subsequent washing five times with PBS to remove the residual ethanol. These steps preceded 6 h of incubation in ATL buffer/proteinase K mix, which was followed by the regular Qiagen tissue DNA extraction and purification procedure as recommended by manufacturer.

### LD-qPCR and LD-RPA assays

The LD-qPCR and LD-RPA assays that were selected have been standardized and evaluated previously for the sensitive and specific detection of *L. donovani* in biological specimens such as blood and skin biopsy obtained from leishmaniasis patients in Bangladesh, as demonstrated in previous reports [19, 26–28]. The LD-qPCR assay was performed by following our published protocol and by using primers and Taqman probe targeting the conserved and repetitive REPL sequence of *Leishmania infantum* (77–142 nucleotides of GenBank accession number: L42486.1) [26, 29]. To estimate parasite load using the LD-qPCR, each run included a standard curve with DNA concentrations corresponding to 10,000 to 0.1 parasites and 1 reaction with molecular-grade water as a negative control. Samples with no cycle threshold value or that ≥ 40 were considered negative. The LD-RPA assay was performed by using kinetoplast minicircle DNA or kDNA (GenBank accession number: Y11401.1) targeted primers and probe, which were developed for the detection of *L. donovani* parasites in clinical samples [19]. Prepared master mix along with the template DNA was placed into tube scanner (Twista, TwistDx, Cambridge, UK) and incubated for 15 min at 42 °C in the only amplification step. The emitted fluorescence signals were measured at 20 s intervals. A combined threshold and first derivative analysis were used for signal interpretation obtained within 15 min.

### Amplicon sequencing

Sequencing was performed to confirm positive detection by LD-RPA assay of LD DNA in samples that were negative in LD-qPCR assay. RPA amplification in triplicates were further incubated for 30 min under the same RPA isothermal condition as mentioned before, followed by accumulation and RPA product purification (PCR Cleanup Kit, New England Biolabs, MA, USA). Thirty nanograms (ng) of purified products from each sample were barcoded using the Oxford Nanopore Technologies' (ONT, Cambridge, UK) Rapid Barcoding Sequencing Kit (SQK-RBK004). Barcoded samples were mixed together followed by ligation of adapter and sequencing by following ONT’s Rapid Barcoding gDNA sequencing protocol (available from https://community.nanoporetech.com/protocols). Libraries produced by the amplicons were sequenced on the ONT MinION using R.9.4 flow-cells and MinKNOW software v3.6.14 (ONT). Raw FAST5 files were base-called using Guppy v3.2.8 (ONT). Reads with a q-score below 7 were discarded and the rest were demultiplexed. For each sample, reads from demultiplexed FASTQ files were aligned to kDNA sequence (GenBank accession number: Y11401.1) using default parameters of Geneious Assembler (Geneious Prime^®^ v2020.1.2) module. The aligned query sequences were then self-assembled into a single contig and thus a consensus sequence was generated that was used as the reference sequence for realignment of all the initial reads. A second consensus sequence was next called from the contig, masking regions below 20× coverage depth. Variants were considered as mismatches and were not further analysed. Finally, consensus coverage of and identity with the primer trimmed reference amplicon (94 nucleotides) within LD-RPA target region of kDNA (160 nucleotides) [19] were calculated after alignment of the consensus with the kDNA sequence by using the default parameters of Geneious Alignment (Geneious Prime^®^ v2020.1.2) module.

### Data analysis

Parametric or nonparametric tests were performed based on the distribution of data. Kappa and McNemar’s tests were performed to determine the concordance and discordance in detection outcome between conventional and molecular methods. Standard statistical formulas were followed to determine the sensitivity and specificity of the tests. All statistical analyses were performed using SPSS (version 20.0, IBM Corp., Armonk, NY, USA) and GraphPad Prism (version 7.03, GraphPad Software, CA, USA). A *P* value ≤ 0.05 was considered statistically significant.

## Results

The main goal of this study was to evaluate LD-RPA and LD-qPCR assays against conventional detection by microscopy, in order to adopt a feasible molecular technique for sensitive detection of sand flies infected with parasites. First, we compared the accuracy of LD-qPCR and LD-RPA against microscopy in detecting LD parasite in reared sand flies, which were fed to PKDL patients. A total of 664 sand flies exposed to the group of 47 PKDL patients were found to be fed, averaging around 14–15 fed sand flies per case. Among these 47 patients, 22 (46.81%) had microscopically observable mobile forms of LD parasites detected in a total of 62/664 (9.37%) fed sand flies. A total of 28 Mic+ sand flies (smeared on slides) representing all the 22 Mic+ infectious PKDL cases were randomly selected from the positive sand fly pool. Following extraction and LD-RPA and LD-qPCR assays, all the 28 sand fly samples were assessed, which resulted in 100% (28/28) and 96.43% (27/28) sensitivity of LD-RPA and LD-qPCR, respectively, and an absolute agreement of either of the methods with microscopy for infectiousness of all 22 patients. None of the apparently unfed sand flies were positive in microscopy or molecular assays, suggesting specific detection of LD parasites in fed sand flies by all methods.

**Fig. 1:**
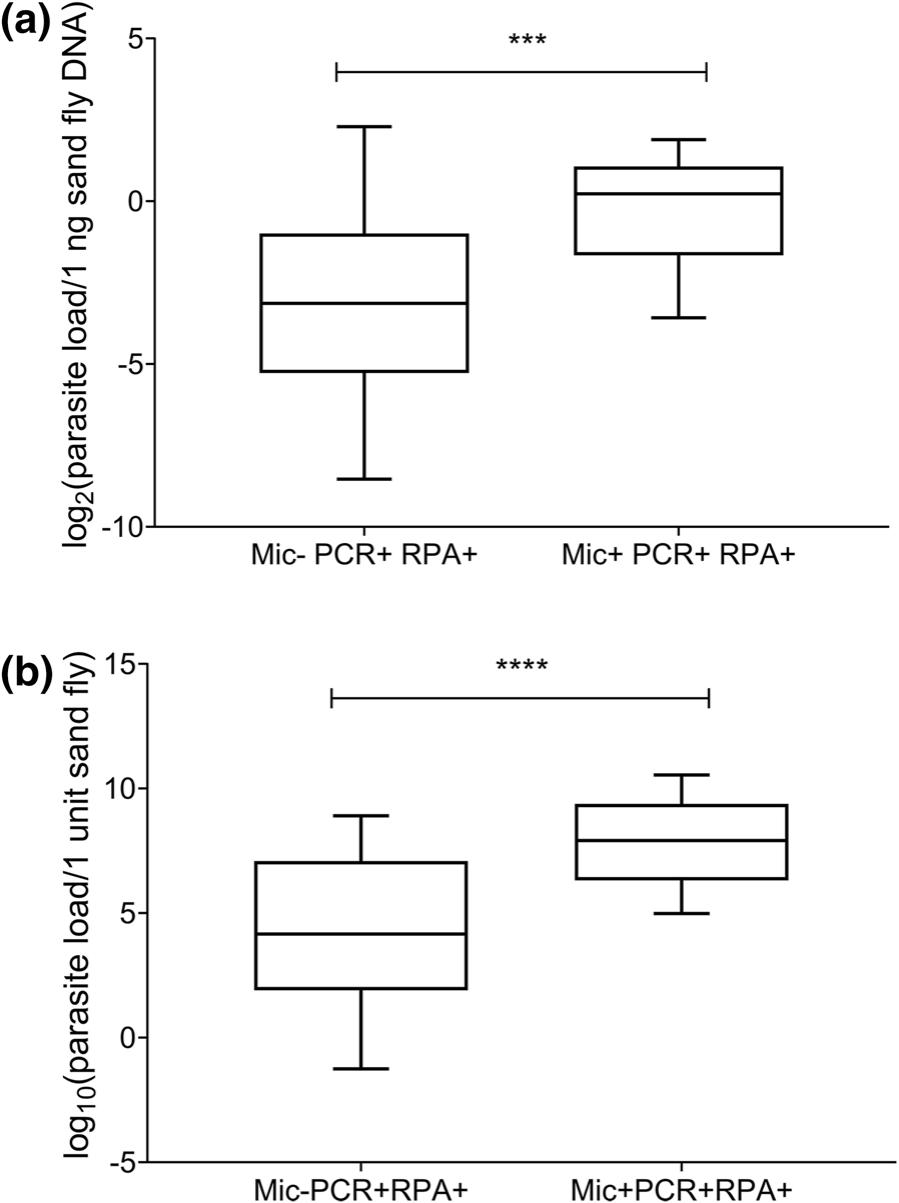
Parasite load (represented in log-transformed value) comparison (Mann–Whitney test, two-tailed, *p*-exact value) at **a** one ng of sand fly DNA and **b** one sand fly (arbitrary unit) levels between microscopy negative but LD-qPCR and LD-RPA positive pooled samples (Mic− PCR+ RPA+;*n* = 19), and microscopy positive smeared samples (Mic+ PCR+ RPA+;*n* = 27). Median parasite loads for Mic− PCR+ RPA+ group and Mic+ PCR+ RPA+ group were **a** 0.11 and 1.17 per ng of sand fly DNA, and **b** 17.89 and 240.4 per sand fly unit, respectively. Box and whiskers plots denote minimum, maximum, median, and interquartile range values. ****p < 0.0001; ***p < 0.001

When Mic− fed sand flies were case-wise pooled and assayed by molecular tools for each of the 47 PKDL cases, it resulted in positive detection of LD in sand fly pools that correspond to 19/47 (40.43%) and 24/47 (51.06%) PKDL cases, as detected by LD-qPCR and LD-RPA assays, respectively. These proportions of cases signified LD detection in Mic− sand fly pools by LD-qPCR and LD-RPA assays of 15/22 (68.18%) and 18/22 (81.82%) PKDL cases, respectively, that had at least 1 fed sand fly positive in microscopy (Mic+). To compare the yield of DNA at unit sand fly level between Mic+ and Mic− groups, extraction performances for Mic+ sand flies (individually smeared, *n* = 28) and Mic− sand flies (case-wise pooled fed sand fly specimens; *n* = 44) were evaluated. Average concentration of DNA yield per sand fly was found to be significantly higher (Mann–Whitney test, *p* < 0.0001, 95% confidence interval [CI]: 126–200) in smeared samples (median: 280 ng; IQR: 260–360) compared to pooled samples (median: 145 ng; IQR: 133.3–193.3), which can be attributed to the differences in DNA extraction methodology and storage condition. On the other hand, evaluation of parasite load could only be performed for the LD-qPCR positive samples (Mic+ smeared sand fly, *n* = 27; Mic− sand fly pools, *n* = 19) from the quantitative PCR amplification data. When extrapolated down to one nanogram sand fly content, it was estimated that the median parasite concentration in Mic+ smear samples (1.17 parasite/ng sand fly DNA; IQR: 0.32–2.1) was also significantly higher (Mann–Whitney test, *p* < 0.001, 95% CI of difference: 0.1675–1.285) than the Mic− pooled samples (0.11 parasite/ng of sand fly DNA; IQR: 0.03 to 0.5). This suggests that the LD-qPCR and LD-RPA assays are capable of detecting much lower parasite load in sand fly samples, which microscopy may be unable to detect (Fig. 1a). The lowest number of parasites estimated in one Mic+ sand fly was 31.56 (median: 240.4; IQR: 79.33–668.4). In comparison, the lowest number of parasites estimated in arbitrarily one Mic− sand fly (averaged from individual pools) was 3.728 (median: 17.89; IQR: 3.728–135.9) (Fig. 1b), which was significantly lower than Mic+ group (Mann–Whitney test, *p* < 0.0001, 95% CI of difference: 65.58–346.8). When respective performance of LD-RPA and LD-qPCR assays on both Mic+ and Mic− samples were combined for overall assessment of proportion of infectious cases, discordance (McNemar test; two-tailed; *p* = 0.04) was observed between the LD-RPA assay (positivity rate: 59.57%; 95% CI: 44.27–73.63%) and microscopy (positivity rate: 46.81%; 95% CI: 32.11–61.92%) results, thus suggesting increased sensitivity of LD-RPA assay in estimating the proportion of infectious cases.

Furthermore, a strong agreement was observed between the molecular tools for identifying infectious PKDL cases. LD-RPA assay was found to detect LD in more smeared and pooled sand fly samples than LD-qPCR, which however was not significant (Table 1). To rule out the possibility of false-positive results, we performed rapid 1D nanopore sequencing assay on the two microscopy and LD-qPCR negative pooled samples which were otherwise positive in LD-RPA assay. The sequencing run generated 45,352 and 25,357 reads, respectively, for the two samples that passed the quality filters (q-score ≥ 7). Alignment of the reads to the kDNA sequence demonstrated full coverage of the amplicon region (94 nucleotides) at 20× coverage depth cut-off (Table 2). The resulting consensus sequences, in which nucleotide variants were considered as mismatches, were 94.8% and 94.9% similar to the primer trimmed amplicon region of reference sequence. The passed reads can be retrieved from https://doi.org/10.5281/zenodo.4293866.

**Table 1:** Comparison among detection methods applied in the detection of LD in sand flies that corresponds to infectious proportion of PKDL cases

Method comparison (as percentage of infectious PKDL cases)		Microscopy	LD-qPCR	LD-RPA
Microscopy negative pooled sand fly specimen only (slide smear DNA excluded)	Positive:	–	40.43%	51.06%
	Negative:	–	59.57%	48.93%
Overall positivity (95% CI) considering smear and pool sample extracted DNA		46.81% (32.11% to 61.92%)	55.32% (40.12% to 69.83%)	59.57% (44.27% to 73.63%)
Discordance and agreement among methods (McNemar test; *κ*-statistic with 95% CI)	Microscopy	–	*p* = 0.5; *κ* = 0.831 (0.675–0.987)	*p* = 0.04; *κ* = 0.748 (0.565–0.930)
	LD-qPCR	–	–	*p* = 0.5; 0.913 (0.796—1.000)
Feasibility of operation	Type of method	Qualitative	Qualitative/Quantitative	Qualitative
	Field applicability	Yes	No	Yes
	Test time acquisition	Individual sample (user-dependent)	Individual sample/pool (105 min)	Individual sample/pool (15 min)
	Detection test cost($)	2–4	30	8–9

**Table 2:** Overview of nanopore sequencing run statistics for LD-RPA amplicon samples

Sample ID	Run length (hours)	Reads generated	Passed reads (%)	Amplicon coverage (%)	Nucleotide consensus similarity (primer-free) (%)	Amplification assay	
						LD-RPA	LD-qPCR
DL-DNA-2-004	8	56,529	49,352 (87.3%)	100	94.8	+	−
DL-DNA-2-014	8	30,027	25,357 (84.4)	100	94.9	+	−

## Discussion

Molecular methods for the detection of genetic material have proven to be more sensitive than biological methods for the detection of *Leishmania* parasites. Consequently, such methods are largely replacing less sensitive parasitological strategies for investigating the host infectiousness, natural history, and epidemiology of leishmaniasis and other transmissible diseases. In the present evaluation, detection of LD parasites in experimental sand fly vectors by molecular tools were compared against conventional microscopy. Our findings suggest that both LD-qPCR and LD-RPA assays have greater potential than microscopy in detecting lower parasite load in sand fly, while LD-RPA can be a rapid and field feasible molecular tool for use in eco-epidemiological and/or xenodiagnostic screening.

Although the molecular methods used in this study (qPCR and RPA) signify only the presence of parasite DNA, its presence is likely associated with the viable and multiplying parasites because exotic DNA taken through a blood meal is generally detectable only within 1–4 days in sand fly and mosquito [30–33]. This is due possibly to the denaturation of DNA during blood digestion [33, 34]. While the minimum parasite load detected in randomly selected Mic+ pool was nearly 32 parasites/sand fly, both molecular tools were found to detect considerably less parasite load arbitrarily in a single sand fly (extrapolated from Mic− pools). However, since molecular tests were performed on DNA extracted from a group of randomly selected Mic+ sand flies and that from Mic− sand fly pools (rather than individual flies), the limits in this comparison may overestimate or underestimate the difference. Because, there could be present more negative flies rather than positive ones in a Mic− pool. Alternatively, the low parasite load might result collectively from parasite-cell-free DNA material that persisted in the sand fly midgut rather than intact parasites. Nevertheless, amplification of two different targets (chromosomal DNA and kDNA) by the two molecular tests in this study perhaps strengthens the argument that molecular tests can render higher sensitivity than microscopy in detecting LD genome in sand fly. This is consistent with a previous observation in which a qPCR assay targeting the kDNA allowed for detection of one *Leishmania* parasite per sand fly midgut, while significant difference in parasite number between qPCR and microscopic observation was also reported during post-infection evaluation of parasite establishment in sand fly [35]. Although we did not test the analytical sensitivity in sand fly in the present study, our previous findings on LD-qPCR assay suggest that its lower limit of detection is 0.1 parasite equivalent genomic DNA [26]. On the other hand, LD-RPA assay has an analytical sensitivity of 1 parasite equivalent DNA, although it can detect copies of target DNA equivalent to less than 1 parasite as well [19]. Both of these tools thus render superiority over microscopy. Interestingly, we observed two LD-RPA positive pooled sand fly samples, which were otherwise negative in LD-qPCR assay as well as microscopy. This is possible since LD-RPA assay targets the kDNA that are abundantly present (~ 10,000 copies per cell) in *Leishmania* species [36]. We further confirmed RPA positivity for those two samples by rapid sequencing of the amplified products of LD-RPA assay followed by evaluation of the amplicon sequence identity with respect to the reference sequence.

The infectiousness of the host is thought to be a crucial driver of transmission in vector-borne diseases [37]. One underlying reason could be the possibility of infecting many sand flies by a single host, who might be infected even only once. However, the observed rate of infectiousness can vary upon the sensitivity of detection method. To our knowledge, this is the first time that RPA-based isothermal method has been explored in detection of parasites in DNA extracted from sand fly vectors that were fed experimentally on PKDL patients. RPA offers crucial advantages including feasibility, expertise, cost, and time over PCR for field deployment of molecular detection, as discussed elsewhere [38]. By using LD-RPA assay as a detection tool, we have found that about 60% of PKDL patients included in this study were infectious to sand fly vector, which is somewhat higher than LD-qPCR (55.32%) and considerably higher than microscopy (46.81%). This is to be noted that our observed positivity for LD-qPCR in this study is higher than that reported in our previous study (44.68%) [10]. This is simply because, unlike in the previous study, we extracted DNA from at least one Mic+ smeared slide per patient by an optimized method as reported here and included the result in the overall infectiousness rate of individual tools. It is thus possible that sand flies were infected disproportionately (i.e., for particular PKDL cases, as low as one sand fly among the fed ones could be the only positive sand fly detected by all methods). Nevertheless, our observed infectious proportion of PKDL cases is higher than that previously observed for American CL (31.3%) [39], but less number and selective reports on experiments involving *P. argentipes* and *L. donovani* in Indian subcontinent [5, 9–11] limits the comparative evaluation for PKDL in this region.

Besides the detection tool, several other factors may come into play for estimation of infection rates in sand flies. For example, skin compartment, coupled with the telmophagic feeding behaviour of the sand fly, may provide a potentially greater source of parasites for sand fly infection than blood in terms of parasite load [40] but less potential in terms of mimicking natural transmissibility [41]. The distribution of parasites in the skin landscape has also been proven as uneven [40]. Another important factor is the severity of the disease. For example, in a previous study on XD of *L. infantum* using its vector fed to dogs, rate of infectiousness to sand flies was found to be positively associated with severity of disease regardless of the method used for parasite detection after XD [42]. However, this may not always be the case; a meta-analysis of dog studies suggested that while the proportion of infectious dogs for sand fly infection may increase with increasing clinical severity, the proportion of sand flies infected by infectious dogs may not vary with clinical status [7]. In our previous study, the association of positive XD of PKDL patients was found mainly with the skin parasite load related to spatial distribution of parasites in the host, i.e., nodular and macular lesions [10]. In this study, we tested further whether factors like the number of sand fly involved in feeding, and the ratios between fed and unfed, female and male, and dead and live sand flies after exposing to the PKDL cases were correlated with positivity in XD by either of the tools. However, no correlation was observed for either of the factors (not shown).

Whether the rate of infectiousness of PKDL cases in sand fly is replicable in natural infection remains to be investigated. Since vector competence differs among phlebotomine species of sand flies [43], required dosage for successful infection may also vary among the *Leishmania* species. In highly susceptible vector species like *P. argentipes* and *P. orientalis*, as few as one or two promastigotes per average blood meal were demonstrated to establish infection in 50% of female sand flies [44]. However, previous reports suggest that a single amastigote replicating this trend would be unlikely [45, 46]. Nevertheless, the minimum amastigote load required for successful sand fly infection is not currently defined. In this aspect, detection by microscopy could be a limiting factor in estimating the infection rates in sand flies, because we found that (as estimated by LD-qPCR) the rate of parasite detection per unit sand fly in Mic+ group can be higher than that (in arbitrary unit) in Mic− group. Although we observed a significantly low concentration of DNA in Mic− pooled samples compared to the smeared samples- due probably to storage and several washing steps during the extraction of pooled samples, parasite DNA-sand fly DNA ratios were still significantly different between the two groups. The higher positivity rates of LD-qPCR and LD-RPA in detecting lower parasite load in experimentally fed sand flies thus suggest a necessity of reassessment of the extent to which PKDL patients play role in the transmission cycle of leishmaniasis. In this context, LD-RPA can be a useful tool for field implementation as a rapid and cheap single molecular LD detection method in sand flies, or as a confirmatory tool for Mic− sand flies. However, the observations presented in this report were based on assessment of a small number of sand fly samples in a laboratory condition, and hence validation of the findings with larger field data would be necessary before exploiting the advantages of the molecular tool.

Despite high sensitivity, parasite load detected by molecular diagnostic tools that amplify DNA targets can be poorly associated with parasite viability [47]. Positive detection following blood digestion, especially of low parasite load, may signify only the persistence or insufficient clearing of parasite-cell-free DNA that resulted from collapsed infection in sand fly during any stage of parasite establishment. One approach to circumvent this limitation can be by targeting parasite RNA as an indirect marker of cellular viability, as RNA degrades fast following cellular death [48]. Since tissue specimens in general are more challenging than liquid samples for the extraction of nucleic acids, further investigation is also needed to standardize a field feasible and rapid method for extraction of nucleic acids from sand fly midgut specimens for the implementation of RPA. Recently we compared magnetic bead-based rapid extraction method coupled with LD-RPA assay for the detection of *L. donovani* in skin biopsy samples of PKDL patients; this however was found underperforming when compared with the reference DNA extraction (column) method using Qiagen kit [49]. In another approach, an extraction method developed by using in-house crude extraction buffer and ethanol precipitation showed similar sand fly DNA extraction efficiencies to column method [48]; however, enhanced centrifugation speed and time could be a limiting factor in its field implementation. Further optimization in nucleic acid extraction and detection parameters would thus be critical for assay sensitivity and accurate estimation of transmissible parasite load in sand flies. In addition, diverse other factors such as skin parasite landscape [40], gut microenvironment [50], and vector permissiveness and competence [51]—alone or combined—may also influence parasite growth and propagation, and hence the sensitivity and usefulness of detection by different tools and should be taken into consideration. For instance, microscopic examination may not be adequately replaced by the molecular tools in the assessment of vector competence in the early phase of *Leishmania* infection in sand flies that are field-caught or fed to experimental host. A successful development of infection in vector is characterized by colonization of the stomodeal valve as well as detection of metacyclic form of parasites [52], and therefore must be checked under the microscope. Furthermore, microscopic examination of a known vector after blood digestion can provide clues as to parasite cellular integrity and maturation in the sand fly which can be associated with onward transmission potential. Future studies should prioritize investigating the limit of molecular detection of parasite in sand fly that renders subsequent transmissibility for effective implementation of such tools in the large-scale investigation of host infectiousness.

## Conclusions

We believe that the presented findings warrant potential field application of RPA-based field-deployable molecular detection method in estimating infection rates in experimental as well as natural sand fly vector in order to understand the transmission trends, and their overall impact on treatment and control strategies. Furthermore, the method can also be implemented as a molecular confirmatory test of LD infection in sand flies.

## Data Availability

Data supporting the conclusions of this article are included within the article. Raw sequence data can be retrieved from https://doi.org/10.5281/zenodo.4293866.

## Abbreviations

PKDL: Post-kala-azar dermal leishmaniasis
LD: *Leishmania donovani*
qPCR: Quantitative polymerase chain reaction
RPA: Recombinase polymerase amplification
VL: Visceral leishmaniasis
KAEP: Kala-azar Elimination Programme
XD: Xenodiagnosis
kDNA: Kinetoplast minicircle DNA
ONT: Oxford Nanopore Technologies
